# The Value of Patient Registries to Advance Basic and Translational Research in the Area of Traumatic Brain Injury

**DOI:** 10.3389/fnbeh.2022.846919

**Published:** 2022-04-25

**Authors:** Melissa C. Duff, Emily L. Morrow, Malcolm Edwards, Ryan McCurdy, Sharice Clough, Nirav Patel, Kimberly Walsh, Natalie V. Covington

**Affiliations:** ^1^Department of Hearing and Speech Sciences, Vanderbilt University Medical Center, Nashville, TN, United States; ^2^Meharry Medical College, Nashville, TN, United States; ^3^Department of Speech-Language-Hearing Sciences, University of Minnesota, Minneapolis, MN, United States

**Keywords:** traumatic brain injury, outcome, registry, intervention, heterogeneity

## Abstract

The number of individuals affected by traumatic brain injury (TBI) is growing globally. TBIs may cause a range of physical, cognitive, and psychiatric deficits that can negatively impact employment, academic attainment, community independence, and interpersonal relationships. Although there has been a significant decrease in the number of injury related deaths over the past several decades, there has been no corresponding reduction in injury related disability over the same time period. We propose that patient registries with large, representative samples and rich multidimensional and longitudinal data have tremendous value in advancing basic and translational research and in capturing, characterizing, and predicting individual differences in deficit profile and outcomes. Patient registries, together with recent theoretical and methodological advances in analytic approaches and neuroscience, provide powerful tools for brain injury research and for leveraging the heterogeneity that has traditionally been cited as a barrier inhibiting progress in treatment research and clinical practice. We report on our experiences, and challenges, in developing and maintaining our own patient registry. We conclude by pointing to some future opportunities for discovery that are afforded by a registry model.

## Introduction

Traumatic brain injuries (TBI) are on the rise globally with 69 million individuals worldwide estimated to sustain a brain injury each year (Dewan et al., 2018). TBIs can cause a constellation of interacting physical, cognitive, and psychiatric deficits that can negatively impact employment, academic achievement, community independence, and interpersonal relationships, leading to long-term disability and poor functional outcomes. While there has been a significant decrease in the number of injury-related deaths over the past several decades, there has been no corresponding reduction in injury-related disability despite considerable efforts in research and clinical spheres (Roozenbeek et al., 2013).

There are a number of factors contributing to the variable and suboptimal outcomes among individuals with TBI. One frequently cited and long recognized obstacle to significant breakthroughs in basic and clinical science is heterogeneity in the population (e.g., Lu et al., 2012). Indeed, TBI is among the most heterogeneous neurological conditions resulting in a wide range of interindividual variability. This variability spans multiple levels of analysis, including wide-ranging differences in premorbid patient characteristics, injury characteristics, pathoanatomic characteristics, and in physical, cognitive, and psychosocial profiles following injury (see Covington and Duff, 2021 for review). Heterogeneity at each of these levels of analysis likely contributes to the highly variable long-term outcomes observed following TBI (Hart et al., 2014; Dahdah et al., 2016). Clinically, heterogeneity observed across individuals with TBI requires the development of personalized treatment protocols. Yet, at the same time, heterogeneity presents a significant obstacle to the development of these personalized treatment protocols for the specific and unique deficit profile of a given patient, and to our ability to predict behavioral and functional outcomes. The issue of heterogeneity in both basic and clinical research is exacerbated by commonly used group study designs with small sample sizes, which do not support analyses that capture factors contributing to interindividual variability in presentation or treatment response.

We recently argued that a shift in strategy is required to advance basic and translational research in TBI, whereby we embrace heterogeneity head on by adopting new methodological and statistical approaches that capture, characterize, and predict individual differences in deficit profile to better assign particular patients to effective treatments (Covington and Duff, 2021). To do this, we must commit to both large, representative samples and to the collection of rich multidimensional and longitudinal data across a variety of contexts and community settings. In the field of cognitive neuroscience, patient registries designed to provide access to large numbers of well-characterized research participants have been highly successful in advancing basic science and cognitive rehabilitation research on the effects of focal and stable brain damage to various cognitive functions (e.g., Damasio and Damasio, 2003; Schwartz et al., 2005; Fellows et al., 2008). We propose that patient registries, inspired by those in the field of cognitive neuroscience, provide a powerful tool for leveraging (rather than being constrained by) the heterogeneity observed in brain injury research and clinical care. To support this proposal, we briefly review the history and value of patient registries in the field of cognitive neuroscience. We describe how a registry approach addresses many long-standing challenges researchers and clinicians face in advancing basic and translational research in the area of TBI. We report on our experiences modifying and extending the registry approach to the study of individuals with TBI (e.g., to support longitudinal and community-based research and treatment designs; collection of repeated measures) and discuss challenges and practical considerations in developing and maintaining a TBI patient registry. We conclude by pointing to some of the unique analytic approaches and future opportunities for discovery that are afforded by a registry model to TBI research.

Before we begin, we should acknowledge that our approach to the challenge of heterogeneity in TBI is from the specific vantage point of understanding cognitive and behavioral disruptions following brain injury and improving rehabilitation and functional outcomes in these domains. Our approach is also through the lens of our unique position in the broader interdisciplinary space that studies and treats individuals with TBI; we are clinician-scientists working at the intersection of cognitive neuroscience, speech-language pathology, and rehabilitation science. From this position, we see an inherent tension between group studies (i.e., that focus on group-level performance or response to treatment) and the clinical reality of treating one individual at a time. A primary motivation of our work is the belief that patient registries can address this tension, are replicable across research and clinical sites, and will improve cognitive and behavioral outcomes following TBI.

Indeed, patient registries of the type we will describe can support both large-scale group studies and community-based individualized intervention research. Following Ylvisaker et al. (2003a), we view TBI as a chronic disability that requires a long-term perspective, deep characterization of individuals long after injury, and a commitment to the development of supports across the lifespan as life with a disability evolves over time. Given this specific perspective, this paper does not offer a complete review of all the sources of heterogeneity that exist in TBI (e.g., mechanisms of injury, psychiatric, and physical impairments) or all the factors that can affect outcome (e.g., type of care, presence, and length of rehabilitation). Rather, we keep our focus on cognitive and behavioral outcomes, although we think the registry approach is equally helpful in understanding the role of these other domains and factors in outcome research. Our goal is to propose the utility of patient registries that focus on understanding individual differences and improving functional outcomes in cognitive and behavioral domains. We also aim to provide a model for establishing a TBI patient registry that is replicable at other research and clinical sites.

## Value of Patient Registries in Cognitive Neuroscience

Knowledge of brain-behavior relationships (i.e., the mapping of cognitive abilities to discrete brain areas) can be traced to a number of individual landmark cases over the past 150 years launching the field of cognitive neuroscience (Koenigs et al., 2007a). Famous case studies led to discoveries about language lateralization (patient “Tan”; Broca, 1865), the role of the frontal lobes in social functioning (Phineas Gage; Harlow, 1868), and the necessity of the medial temporal lobes for declarative memory (patient HM; Scoville and Milner, 1957). Over the following decades, other single case studies were added to the literature providing additional evidence for proposed brain-behavior relations (e.g., medial temporal lobes and memory; Squire and Moore, 1979; Damasio et al., 1985) and new discoveries (e.g., amygdala and emotion; Adolphs et al., 1994). While the study of single cases, known as the lesion method, has remained a cornerstone of cognitive neuroscience research (Damasio and Damasio, 2003; Adolphs, 2016; Vaidya et al., 2019), criticisms of the approach have included concerns regarding generalization and reproducibility of findings. The argument was that lesion dimensions and cognitive presentation of single patients are too variable, or idiosyncratic, to be informative or replicated (e.g., because single cases vary across factors such as age, education, sex, and intelligence; because lesions cannot be experimentally produced in humans).

These criticisms have largely been addressed by assembling larger groups of patients who are well characterized demographically, neuroanatomically, and neuropsychologically. Larger group studies of patients with stable and focal brain lesions allowed researchers to conduct hypothesis-driven research about the necessity of particular brain regions for particular cognitive abilities. These larger samples increased experimental control of known sources of variability and permitted conclusions based on participant groups that are comparable in size to traditional psychological experiments (Koenigs et al., 2007a). Damasio and Damasio (2003) have written about prerequisites for optimal practice of the lesion method in group studies, including careful participant characterization, use of valid and reliable experimental measures, and a commitment to collecting multidimensional data. These sources of multidimensional data include: (1) detailed structural imaging data of the human brain *in vivo* and reliable methods for neuroanatomical analysis; (2) reliable measurements of cognition and behavior (i.e., neuropsychological assessment, experimental tasks); (3) a large pool of participants from which to form an experimental group (with target lesion) and a comparison group (with damage outside the region of interest or no neural damage at all); and (4) demographic-matching of the experimental and control groups, in order to remove the confounding influence of factors such as age and education.

The need for a larger pool of well-characterized participants, who can be grouped on various dimensions (e.g., lesion size, location) and then matched to a comparison group, led to the formation of patient registries (e.g., University of Iowa, University of Pennsylvania, McGill University; Damasio and Damasio, 2003; Schwartz et al., 2005; Fellows et al., 2008). These registries, particularly the Patient Registry of the University of Iowa’s Division of Behavioral Neurology and Cognitive Neuroscience, have produced a number of discoveries regarding the neural correlates and brain-behavior relationships in the domains of language, memory, and emotion that have significantly advanced the field of cognitive neuroscience (e.g., Adolphs et al., 1994; Bechara et al., 1995; Tranel et al., 2003; Damasio et al., 2004; Koenigs et al., 2007b).

An important observation that comes from these group studies of focal lesion patients is that with sufficient numbers and carefully constructed and matched groups, behavioral performances of the target participants (i.e., individuals with damage to a shared and specific neural region) are often quite similar and lack the variability or idiosyncratic performances for which individual case studies have been criticized or discounted. That is, there is tremendous heterogeneity in behavioral performances when all individuals with a focal lesion are grouped together. Yet, when meaningful subgroups of participants can be identified (i.e., based on shared presence of a brain lesion in an area of interest), this heterogeneity is reduced and we uncover factors that predict *behavioral* outcome (e.g., medial temporal lobe lesions are associated with memory deficits). Wide sampling, together with the collection of multidimensional data, also provides the opportunity to observe individual differences in *functional* outcome. For example, medial temporal lobe lesions are consistently associated with memory deficits. Yet, individuals with shared medial temporal lobe damage and comparable severity of memory deficits can vary in their functional outcomes (i.e., independence, employment, satisfaction with life) leading to novel, testable hypotheses about the role of various factors (e.g., age at injury, sex) in functional outcomes (Duff et al., 2008; Warren et al., 2012).

Given the goals of the lesion method (i.e., to test hypotheses about the role of a focal neural region in supporting a specific cognitive function or behavior), patient registries in cognitive neuroscience have focused on recruiting and studying individuals who have a single, focal, and stable (or non-progressive) lesion to gray matter. Thus, individuals with TBI (who have diffuse patterns of neuroanatomical damage, affecting multiple regions of gray and white matter) were not appropriate participants for, and have been excluded from, traditional patient registries conducting focal lesion studies. Yet, we believe that some of the features of the lesion method and patient registries in cognitive neuroscience can be strategically adapted and extended to support methods capable of leveraging the heterogeneity observed in brain injury research and clinical care. Further, it is our assertion that patient registries, together with recent theoretical and methodological advances in statistical analysis and cognitive neuroscience, offer the tools necessary to improve our understanding of individual differences in behavioral profiles and functional outcomes which can inform the development of personalized interventions to improve long-term outcomes.

In the next sections, we review some existing barriers in TBI research and propose ways a registry approach could be a transformative tool for advancing the study and clinical management of individual differences in long-term behavioral outcomes following TBI. We then describe practical considerations in developing and maintaining a TBI patient registry and outline challenges we have faced along the way. We conclude by pointing to some future opportunities for discovery that are afforded by a registry model.

## Value of Patient Registries for Advancing Basic and Translational Science in the Area of Traumatic Brain Injury

Patient registries inspired by those in cognitive neuroscience, which support the recruitment of large representative samples and the collection of multidimensional data, can provide a powerful tool for leveraging the heterogeneity observed in brain injury research and clinical care. To support this claim, in this section, we describe significant challenges in advancing basic and translational research in TBI and some initial ways a registry approach, together with more recent theoretical and methodological approaches in statistics and cognitive neuroscience, could be a powerful tool for conducting large scale empirical studies that (1) capture and better characterize sources of interindividual variability that contribute to cognitive and behavioral profiles and outcomes, and (2) track the diverse experiences and challenges of living with a brain injury over the lifespan, with the long-term goal to (3) inform the development of meaningful subgroups of individuals with TBI and the identification of factors that influence individual differences in behavioral and functional outcomes in TBI.

Before moving on we should acknowledge existing large-scale data sets in TBI research [e.g., TBI Model System (Dijkers et al., 2010), for review; state level epidemiological registries], that at first blush can resemble the type of patient registries we are discussing here, but that differ in key ways. First, many existing TBI data sets are *broad* (i.e., collect data from many participants), but *thin* as they often collect a single measure per cognitive domain, self-reported outcome, or injury characteristic. Registries in cognitive neuroscience, and those we will advocate for below for TBI research, collect data sets that are both broad and deep: multidimensional demographic, neuroanatomical, and neuropsychological data with multiple measures per construct of interest for rich characterization and hypothesis testing (see Damasio and Damasio, 2003; Tranel, 2009). Second, many existing large-scale data sets follow individuals closely from the acute phase of injury through in-patient rehabilitation and then intermittently over time. These data sets provide a critical understanding of the first years of recovery for those with access to rehabilitation services. Less is known about how individuals with TBI and variable access to rehabilitation services navigate living with a chronic disability, or how they reintegrate into vocational, community, and interpersonal spheres over time. A focus on individuals with the need and access to in-patient rehabilitation can bias sampling on the basis of behavioral severity and socioeconomic variables. Finally, a significant strength of existing TBI data sets has been their scale. Some programs have multiple satellite centers across the country, pooling data to increase sample size and geographic diversity. Existing large-scale data sets in TBI research have provided important insights about TBI as a group, but we still know less about individuals with TBI. A rehabilitative focus with increased attention to individual differences and individualized treatment moves our research beyond knowing whether a given treatment effect exists for the “average person” with TBI to begin to predict individual differences in deficit profile that might lead to better assignment of particular patients to specific treatments. Furthermore, research registries within local communities, or a satellite center, offers a unique opportunity to conduct personalized treatment studies situated in the specific communities and spaces where an individual lives and works and to better understand the contextual and environmental factors that can promote or impede generalization of treatment outcomes to everyday settings for the individual.

Below, we argue for longitudinal designs and experimental studies of diverse individuals with TBI, starting shortly after injury and continuing deep into the chronic epoch of injury. We also advocate for a greater focus on individual differences and individualized rehabilitation research. Thus, the type of patient registry we propose below offers a complementary, yet novel and translational approach that can be replicated by other groups working to advancing rehabilitation and long-term outcomes in TBI.

### Behavioral Profile Characterization

Heterogeneity in behavioral profiles is a frequently cited and long recognized obstacle to significant breakthroughs in advancing basic and clinical science in the area of TBI (e.g., Lu et al., 2012). Indeed, as a diagnostic category, traumatic brain injury is, at best, descriptive only of the *mechanism* of injury. But even the mechanisms and circumstances of injury are variable, with differing sources and severities of impact resulting in diverse patterns of pathoanatomic characteristics with variable profiles of both focal and diffuse lesions (Hawryluk and Manley, 2015). While patients with TBI do share commonalities, under this broad diagnostic category they vary widely in initial injury severity, and in patterns of strength and weakness across cognitive domains. At all levels of analysis, TBI results in striking variability. Individuals who sustain a TBI differ in their premorbid characteristics, many of which (age, sex, education) have been shown to impact patient outcomes (Senathi-Raja et al., 2010; Lah et al., 2011; Schneider et al., 2014; Mollayeva et al., 2018; Turkstra et al., 2020). Finally, individuals with TBI present with a range of cognitive and behavioral deficits with variable severity of impairment across domains and heterogeneous behavioral profiles. This multifaceted heterogeneity has been cited as a primary hindrance to progress in characterizing, assessing, and treating cognitive dysfunction post-TBI.

One traditional approach in TBI research has been to recognize this variability in behavioral profiles and attempt to constrain it by recruiting only those individuals with a particular profile at a particular level of analysis. This is the opposite approach taken by lesion method registry studies where recruitment is not constrained by behavioral or functional outcome but by the presence of a lesion (or for our purposes, a diagnosis of TBI). Yet, across TBI studies, many groups and labs use differing inclusionary and exclusionary criteria based on limited information about the individual —making comparisons of results across studies a significant challenge. We should note that there have been multiple attempts to reduce this variability in classification and data reporting [e.g., NIH Common Data Elements for TBI (Maas et al., 2010), Mayo Classification System for Traumatic Brain Injury Severity (Malec et al., 2007), The Management of Concussion-mild Traumatic Brain Injury Working Group (2016)]. Adoption of these guidelines, or reporting of their use, however, remains inconsistent across subdisciplines of the field, perhaps due to differences in setting, research focus, and funding sources.

Furthermore, a large proportion of studies in basic and translational TBI research have employed case-control analyses. This design decision ignores multilevel heterogeneity in the TBI population, and compares single group-level point estimates. Often, this means that meaningful interindividual differences are averaged out. Exacerbating the problem are the small sample sizes that are typical within the field. In the context of a high degree of heterogeneity and small sample sizes, contradictory group-level findings are inevitable (see Lombardo et al., 2019 and Covington and Duff, 2021 for discussion). Meaningful subgroups within the larger population may be sampled to differing degrees in two studies examining similar research questions, resulting in conflicting conclusions. Even in cases where the study sample adequately represents the larger population, if there are meaningful subgroups within the larger sample, these individual differences are lost in group-mean designs. Particularly for treatment and rehabilitation studies, these underlying individual differences may result in non-trivial differences in response to intervention (Kent et al., 2010).

We have argued that attempts to address heterogeneity by constraining study samples misses the point (Covington and Duff, 2021). TBI is *inherently* heterogeneous, and we don’t yet have enough information to determine *a priori* what aspects of that heterogeneity are useful for subdividing the larger population into meaningful groups. For example, through the efforts of wide and inclusive sampling, lesion method registry studies revealed that subdividing the larger population of individuals with focal lesions by lesion location (rather than by outcome) proved to be a meaningful factor in establishing and predicting behavioral outcomes (e.g., medial temporal lobe lesions are associated with memory deficits). Note, however, that grouping by lesion location and behavioral deficit does not reliably predict *functional* outcomes (i.e., not everyone with a medial temporal lobe lesion and a profound memory deficit has the same level of independence, employment, or satisfaction with life). This suggests that predicting functional outcomes requires a different set of grouping variable(s) (e.g., age at injury, sex, extent of white matter damage, level of family support), an approach made possible with the collection of multidimensional data on each individual.

We do, of course, have some information about variables that predict outcomes. Injury severity [as measured by the Glasgow Coma Scale (GCS)], length of post-traumatic amnesia, and location and extent of neural damage have all been shown to predict functional outcomes at the group level (e.g., Kim, 2011). For example, research shows that higher GCS scores are associated with better functional outcomes than lower GCS scores. But every clinician has had the experience of observing a patient with a GCS of 3 go on to have positive functional outcomes in community reintegration and vocational and interpersonal pursuits and a patient with a GCS of 15 struggle with negative functional outcomes years after injury. Thus, severity is a good predictor of outcome for the group, but less predictive for the individual. We need to better understand the other factors that contribute to these individual differences, alone or in combination with a single measure like GCS.

Instead of bemoaning heterogeneity and designing group studies that attempt to strictly control it, we argue that an approach that *embraces* the inherent heterogeneity in the population of individuals with TBI is more likely to yield fruitful results. Rather than artificially constraining the sampled population to a particular pre-defined subset of the larger population of patients with TBI, we suggest attempting to sample across the real-world range of neuropathologic and cognitive profiles, and committing to large sample sizes and to collection of multidimensional data. Doing so opens up a wide array of possible analytical approaches (described in section “Analytic Approaches and Future Opportunities for Discovery Afforded by a Registry Model,” below) that attempt to parse and explain heterogeneity in meaningful ways.

Research that leverages the heterogeneity following TBI could advance the field by identifying factors that influence individual differences in behavioral and functional outcomes, and could establish meaningful subgroups for matching individuals to effective interventions. The challenge is that we don’t yet know what aspects of that heterogeneity are meaningful for the group vs. the individual. Thus, well intentioned and commonly deployed research designs (including ours) that attempt to constrain heterogeneity through recruitment, inclusion, and analytic approach may actually be impeding progress and making it more difficult to identify factors that predict outcomes. We propose applying and extending methods used by patient registries in cognitive neuroscience to address this challenge.

### Neuroanatomical Characterization

Neuroanatomical pathology is another significant source of heterogeneity that affects cognitive and behavioral outcomes. The initial source of injury variably impacts the brain, with notable differences between injuries caused by linear versus rotational acceleration that produce varying degrees of secondary consequences during acute medical care. Once the cascading effects of secondary sequelae have stabilized, the resulting pattern of pathoanatomic characteristics yields variable profiles of both focal and diffuse lesions (Hawryluk and Manley, 2015). A patient’s resulting stable pathoanatomic profile, however, does not directly or simplistically correspond to a cognitive or behavioral profile (Bigler, 2007; Saatman et al., 2008). A registry approach that affords the rich behavioral data, combined with the latest advances in anatomical and functional neuroimaging, may help to disentangle multilevel sources of heterogeneity and better link neuroanatomical profiles to cognitive abilities and behavioral outcomes.

In TBI, gray matter damage is common. However, it is the widespread damage to white matter tracts, or diffuse axonal injury (DAI), that is the defining neuropathological consequence of TBI —cutting across all injury severities (Adams et al., 1989; Bigler, 2007; Gentleman et al., 1995). In fact, the DAI has led some to suggest that TBI may be best understood as a disconnection syndrome, in which white matter lesions cause network disruption at structural and functional levels (Irimia et al., 2012; Hayes et al., 2016). The lesion method, and neuroscience more broadly, has historically focused on gray matter, and patient registries established to study brain-behavior relations have thus excluded individuals with TBI. Yet, more recent work points to the critical role of white matter in complex cognitive functions as well as for understanding dysfunction across clinical populations (e.g., Wang and Olson, 2018).

Until relatively recently, DAI and white matter damage had been difficult or impossible to quantify in-vivo. Early neuroimaging methods, such as CT and structural MRI, were unable to detect subtle micro-anatomic lesions. With the advent of diffusion tensor imaging (DTI) and functional magnetic resonance imaging (fMRI), there has been increased interest in the study of structural and functional connectivity amongst neural structures and in comprehensive characterization of the neuroanatomical pathology across gray and white matter structures following TBI (Adams et al., 2000; Kinnunen et al., 2011; Hellyer et al., 2013). These advanced methodological techniques, together with a registry approach focused on multidimensional and longitudinal data from large samples of participants with TBI, promise not only to be a powerful tool for characterizing the neuroanatomical correlates of behavioral change and outcome in TBI but may also advance basic neuroscience more broadly.

For example, neuroimaging research over the past several decades now suggests that there is not a “one-to-one” mapping of a single cognitive ability to a discrete neural region. Rather, multiple neural systems can contribute to a cognitive ability. Conversely a single neural system can make contributions to multiple cognitive functions. Accordingly, cognition is now widely viewed as being organized around large-scale networks comprised of hubs (regions of gray matter) and the connections between hubs (white matter). Network neuroscience functions conceptually by treating large-scale brain networks as groupings of simplified region-to-region relationships where structural and functional connectivity is dynamic and can be characterized and quantified (e.g., Bressler and Menon, 2010; Bassett and Sporns, 2017; Barrett and Satpute, 2013; Sporns, 2014). This approach has been informative in identifying network disruptions in a number of brain disorders, including TBI (Pandit et al., 2013; Crossley et al., 2014; Hayes et al., 2016; Bassett et al., 2018; van den Heuvel and Sporns, 2019; Bilger and Allder, 2021).

Traditional lesion method studies of individuals with focal lesions have expanded to embrace this network approach (e.g., lesion-network mapping; Sutterer and Tranel, 2017) and have integrated prior findings with new discoveries to test both local and network hypotheses, advancing our understanding of complex behavior (Vaidya et al., 2019). A striking example of the power of combining a registry approach with network neuroscience comes from a study of over 500 participants with a focal lesion (> 400 from the Iowa Patient Registry) and rich multidimensional neuroanatomical and neuropsychological data (e.g., cognitive assessments spanning multiple cognitive domains) (Reber et al., 2021). The study authors reported that damage to white matter is linked to worse cognitive outcomes, whereas gray matter lesions were less consistently associated with cognitive outcomes. This approach highlights the critical role of white matter in supporting cognitive outcomes, but may also be helpful in future attempts at explaining interindividual differences in cognitive and behavioral outcomes following diffuse brain injury.

Network-based models hold promise for advancing TBI research by establishing associations between white matter damage and cognitive and behavioral disruption. In our own work we have demonstrated correlations between performance on both facial affect recognition and face-based theory of mind tasks with specific patterns of disruption in long range association fibers (Rigon et al., 2018; Edwards et al., 2019). Other work shows relations between white matter integrity in TBI and information processing speed, executive functioning, learning, memory, and functional outcome scores (Kinnunen et al., 2011; Spitz et al., 2013; Strangman et al., 2012; Caeyenberghs et al., 2014). Thus, whereas diffuse neural pathology and widespread cognitive impairment were exclusionary factors in traditional lesion method registry studies, consideration of cognition from a network perspective affords unique opportunities to leverage interindividual neuroanatomical differences in TBI to test hypotheses about how large-scale networks support cognition and behavior.

To date, however, network-based approaches in TBI have focused largely on identifying behavioral pathology at the group level —drawing correlations between behavioral pathologies and specific patterns of white matter disruption or specific changes in functional connectivity within and/or between networks. As noted previously, this design decision tends to result in meaningful individual differences being averaged out. Similar to behavioral studies, small sample sizes may result in uneven sampling of meaningful subgroups within the TBI population—resulting in contradictory group-level findings. In sufficiently large and representative samples supported by patient registries, like the Reber et al. (2021) study described above, future TBI studies could apply network-based models to examine variable interindividual patterns of deficit, an approach that may also provide a powerful tool to identify patient subgroups with shared patterns of neural damage related to cognitive deficit profiles and for predicting behavioral and functional outcomes (see section “Analytic Approaches and Future Opportunities for Discovery Afforded by a Registry Model,” below).

### Tracking, Monitoring, and Improving Long-Term Outcomes

Heterogeneity in long-term functional outcomes, including return to employment, independent living, and opportunities for social activity, are well documented in TBI (Sander et al., 2010). A significant challenge in improving outcomes at the level of the individual is that we have an incomplete understanding of the critical factors that moderate recovery and treatment efficacy. Here too, we propose that patient registries offer a unique opportunity to address this gap in the literature. In addition to the collection of multidimensional data to better disentangle multilevel sources of heterogeneity, an additional strength of patient registries is that they can provide the necessary infrastructure for long-term tracking and monitoring of chronic injury-related disability in a given community. Registries and other tools that support longitudinal data collection have been identified as a key research need and opportunity in the field to advance long-term outcomes (Interagency Committee on Disability Research, 2016; Haarbauer-Krupa et al., 2021).

Traumatic brain Injury is a chronic disability, yet the modern medical system treats it as an acute injury (Ylvisaker et al., 2005; Katz et al., 2006; Dahdah et al., 2016; Morrow et al., 2021). Rehabilitation services (e.g., speech, physical, and occupational therapies), when available, are often front-loaded for intensive therapy courses in the weeks or months post-injury. As a consequence, some patients, due to limitations in access or insurance, may not receive rehabilitation services beyond their initial hospitalization (Ylvisaker et al., 2003b,Ylvisaker et al., 2005). Providers who see patients during the acute stages of TBI must prepare those patients to live with a long-term disability despite limited tools to predict the nature of that disability, to target services to a given individual, or to provide support to chronic patients over time (Dahdah et al., 2016; Morrow et al., 2021). Thus, not only are we limited in our knowledge of what the long-term challenges are that a given individual will face when returning to home and work, we do not have systems in place to follow individuals with TBI over time to obtain this information and provide directed, personalized interventions to address these challenges as they arise.

While cognitive and neuropsychological profiles are stable in the chronic phase of injury (Salmond et al., 2006), injury-related disability in TBI can change over time (Ylvisaker et al., 2005; Dahdah et al., 2016; Morrow et al., 2021). Long-term disability post-TBI is dynamic, driven in part by difficulty with flexible and adaptive behavior in response to changing contexts and life circumstances (Ylvisaker et al., 2003a; Katz et al., 2006). Some patients, having managed a smooth return to a well-known job or home environment after injury, may be unable to adapt to changes to that environment (e.g., a new system to learn at work) that occur months or years later. Individuals with TBI may struggle over the course of their lifetimes to adapt to everyday struggles or major life events (e.g., moving house, changing jobs, death in the family; Ylvisaker and Feeney, 1998). This difficulty with flexible and adaptive behavior may underlie the unpredictable and fluctuating nature of disability post-injury and may partially explain why individuals with a history of TBI are at risk for a variety of negative outcomes, including social isolation (Rigon et al., 2019a), unemployment (Keyser-Marcus et al., 2002; Ownsworth and McKenna, 2004; Gormley et al., 2019), housing insecurity (Stubbs et al., 2020), interactions with the legal system (McIsaac et al., 2016), interpersonal violence (Ivany and Schminkey, 2016; Valera, 2020), and repeat injury. Research based exclusively on single-timepoint tasks in the lab or clinic may inadequately capture how cognitive disability manifests across contexts and in real-world settings.

Patient registries can be a critical tool in addressing these gaps (Haarbauer-Krupa et al., 2021). Developing relationships with patients and monitoring their performance over time surpasses single-timepoint studies by allowing for repeated, dynamic assessment of performance and outcomes. We have used our Registry to develop lines of research that begin to tackle critical gaps by following a large group of patients with chronic TBI over different time scales. For example, we have used short-term longitudinal designs to assess how learning of new words evolves over the course of weeks, rather than during a single visit (Morrow et al., 2022). Conducting studies like this in large samples of well characterized participants also affords the opportunity to examine individual differences in performance over time. Together with other sources of multidimensional data, short-term longitudinal designs allow us to start asking questions about which individuals, or groups of individuals, benefit from specific interventions.

Patient registries also afford the infrastructure to obtain repeated measurement on the experiences of people with TBI and the response to changing life or environmental circumstances. Current clinical and research structures that do not encapsulate long-term follow-up are inadequate for capturing the evolving nature of TBI-related disability over time, and often fail to respond to individual needs that could ensure high levels of community integration and participation. In our own work, we collect data over time on employment, housing, satisfaction of life, and community participation. We have also surveyed respondents to assess their use of compensatory memory aids (Covington et al., 2021) and their experiences during the COVID-19 pandemic (Morrow et al., 2021). In the case of our pandemic survey, our Registry allowed us to conduct research that was responsive to world events requiring flexible and adaptive behavior and to collect first-person accounts from individuals with TBI about how those events affected them in real time, which then drove the design of follow-up studies. This work is dependent on established relationships with individuals with TBI through their participation in the Registry and falls in line with calls for increasing participatory research with input from individuals with TBI themselves (Ehde et al., 2013). Access to well characterized participants who participate in research studies over time provides a critical tool for understanding the lived experiences of patients with TBI and for using those experiences to inform research that matters to patients, to advocate for critical topics in public health messaging, and to educate the broader public about the implications of TBI over the lifetime (Ehde et al., 2013).

### Summary

Our proposal that heterogeneity be embraced rather than avoided parallels calls from diverse fields to leverage heterogeneity in both mechanistic and treatment research (in behavioral science Bryan et al., 2021; autism; Lombardo et al., 2019; medicine; Del Prato, 2019; psychiatry; Insel, 2014). Each of these proposals echoes the need for both large representative samples and for rich longitudinal characterization of participants, in order to uncover the moderating factors that drive variable outcomes and response to intervention. Long-term, the establishment of such data-rich resources allow for research designs and statistical techniques capable of decomposing and understanding meaningful variability in patient profiles. We argue a patient registry model is best able to support this type of data collection.

## Practical Considerations for the Establishment of a Traumatic Brain Injury Patient Registry

Here, we describe our approach to developing and maintaining a patient registry modeled after registries in cognitive neuroscience but adapted to meet the specific challenges of studying TBI. A goal of our work is to provide a model for establishing a TBI patient registry that is replicable across research and clinical sites by other groups working to understand individual differences and to advance rehabilitation and long-term outcomes in TBI. Our registry, The Vanderbilt Brain Injury Patient Registry, is modeled after and builds on the Patient Registry of the University of Iowa’s Division of Behavioral Neurology and Cognitive Neuroscience. The first author trained and worked at Iowa gaining experience in the lesion method with individuals with focal brain injuries. This 11-year long experience provided significant insights into procedures and best practices for maintaining the necessary infrastructure to support a highly successful patient registry. In this section we report on our experiences in extending a lesion method registry for the study of TBI and in developing and maintaining our TBI patient registry, established first at the University of Iowa and now at Vanderbilt University Medical Center.

The long-term goal of the Registry is to capture, characterize, and predict individual differences in deficit profile and outcomes in order to better assign particular patients to effective treatments. In developing our TBI registry, we were guided by best practices identified by Damasio and Damasio (2003; also see Koenigs et al., 2007a; Tranel, 2009) including a commitment to multidimensional data for participant characterization (e.g., demographic, neuropsychological, neuroanatomical). In the same way, our Registry is committed to wide participant sampling to ensure a sufficiently large and representative sample. We also aim to develop valid and reliable experimental designs, to deeply characterize the neuropsychological and neuroanatomical profile of each enrolled participant, and to recruit comparison participants to support demographic-matching for large group studies addressing basic science and translational research questions. Finally, we aim to create a research infrastructure that maintains the long-term retention of participants to support single-time point studies as well as short-term and long-term longitudinal studies for tracking and monitoring outcomes over time.

### Types of Data Collected

#### Patients With Traumatic Brain Injury

The Registry is structured to collect multidimensional data on each enrolled participant. Neuropsychological data collection follows the Iowa-Benton method (Tranel, 2009) which starts from a core battery that evaluates: (1) intellectual abilities; (2) memory; (3) speech and language; (4) perception and attention; (5) visuoconstruction ability; (6) motor function; (7) executive function; and (8) personality and affect. There are multiple measures per domain (e.g., memory; Rey Auditory Verbal Learning Test, Complex Figure Test) and several assessments recommended as common data elements for traumatic brain injury research (Maas et al., 2010) are part of the core battery (e.g., Auditory Verbal Learning Test; Trail Making Test). The majority of these assessments have well-established psychometric properties and normative data. Many of the assessments in the battery are also commonly employed in the clinic, which increases the interpretability of our findings.

We also supplement our core neuropsychological battery with experimental measures. For example, in addition to traditional neuropsychological tests of memory in the core battery (e.g., Complex Figure Copy, Auditory Verbal Learning Test), participants also complete experimental tasks assessing declarative memory in tasks that place increased demands on relational processing (e.g., memory for face-scene pairs, Morrow et al., 2020; memory for spatial relations, Rigon et al., 2019b). Experimental measures allow for data collection methods beyond traditional neuropsychological assessment and may better capture core deficits common in TBI (e.g., difficulty integrating sources of information in the moment, difficulty completing tasks in less-structured environments). Finally, we also collect data regarding community integration, quality of life, and other self-report measures (e.g., Sydney Psychosocial Reintegration Scale; Tate et al., 2011), to capture aspects of the lived experience of TBI that may be internal or socially situated and may be missed in formal assessments. These measures also allow us to link more “objective” but impairment-based measures (e.g., standardized memory assessment) to patient-reported long-term outcomes (e.g., participation, social reintegration).

Neuroanatomical data collection for the Registry mirrors the traditional goal of linking brain-behavior relationships of prior cognitive neuroscience patient registries while also taking advantage of theoretical and methodological advances in network neuroscience. Neuroimaging methods include magnetic resonance imaging and DTI for structural quantification of gray and white matter, respectively, and resting state fMRI to evaluate functional connectivity of distributed brain regions. Our lab has a particular interest in the medial temporal lobes and the hippocampus, structures particularly vulnerable to injury mechanisms common in TBI. We are planning to soon start collecting magnetic resonance elastography, a non-invasive assessment of viscoelastic mechanical properties of the human brain, which has been shown to have increased sensitivity to behavioral performance than volumetric analyses (Schwarb et al., 2017).

#### Non-injured Comparison Participants

In general, non-injured comparison participants enrolled in the Registry do not complete the full battery of assessments and tasks. Many of the neuropsychological assessments include normative data from the general public. In contrast, for experimental measures, for which normative data are not available, we always recruit non-injured comparison participants who are demographically matched (e.g., on age, sex, level of education, handedness) to participants with TBI. In addition to these experimental measures, it is ideal to have some cognitive or neuropsychological data on non-injured comparison participants for additional matching or for use as independent variables/covariates. For this purpose, depending on time and other study-specific goals, we have sometimes used a quick and broad standardized tool (e.g., NIH Toolbox) that is administered to all participants and other times have administered a subset of assessments from the full neuropsychological battery to the comparison participants.

### Recruitment and Retention

The success of a patient registry depends on recruiting and retaining a large and diverse pool of well-characterized participants. We recruit participants with TBI across all severity levels (mild, moderate, severe) and across the continuum of care and the lifespan. To reach the largest number of potential participants and to recruit as diverse a sample as possible, we use a variety of recruitment strategies. The most effective approach has been recruitment from medical records of individuals with a history of TBI at our medical center. Individuals with a TBI diagnosis are sent mailers describing the Registry and inviting them to contact us if they are interested in participating in research. A research nurse searches for new medical records every 4–6 months. We also recruit potential participants using social media ads, community fliers, and clinician word of mouth. Evaluation of our recruitment strategies is iterative as we determine which methods are effective (e.g., we have had poor luck with newspaper and bus ads). We invite participants whether or not they received any in-patient or outpatient rehabilitation. Developing high-quality materials that explain the Registry and that can be distributed to potential participants and providers in the community has also been helpful in recruiting participants.

A critical support for the Registry is the Clinical and Translational Research Coordinator. This position is best filled by someone with experience working with individuals with TBI and who shares in the long-term goals and mission of the Registry, increasing the likelihood they will be a long-term team member. The Research Coordinator plays a pivotal role in recruitment and retention and in building sustained relationships with participants, investigators, and community stakeholders. Our Coordinator conducts an extensive structured interview with participants to obtain information about medical, academic, vocational, and social history. They also review medical records to obtain injury-specific medical information and to determine TBI severity using published methods (Malec et al., 2007). Recruitment into the Registry is inclusive. We do not exclude participants based on age at injury, a history of multiple injuries or a history of significant psychological or drug abuse history (though these are common exclusionary criteria for group-design studies in the field). We note this information in our database, together with demographic and injury related information, so that individual investigators can select potential participants for a given study based on their specific inclusion and exclusion criteria and to form appropriate groups for hypothesis testing. The Clinical and Translational Research Coordinator then serves as the liaison with investigators to assist in searching the database of potential participants, forming appropriate group matches (i.e., matching each participant with TBI to a non-injured comparison participant with the same age, sex, and educational attainment), and in inviting and scheduling participants for studies. Recruitment is a time-intensive and sustained activity of a registry. Since starting our Registry at Vanderbilt in 2017, we have enrolled 156 individuals with TBI and 213 non-injured comparison participants.

Larger samples alone are insufficient to advance TBI research. Recruiting a participant pool that is diverse in terms of representation across race, ethnicity, gender, sex, socioeconomic status, and geography (rural vs. urban) is critical to advancing research on long-term outcomes and also in understanding and addressing health inequities following TBI. For example, racial and ethnic disparities in outcomes following TBI exist at all points along the continuum of care (Saadi et al., 2021) and minority groups exhibit lower levels of community integration and lower levels of satisfaction with community, civic, and leisure participation following their injuries than White TBI survivors (Mascialino et al., 2009). While our recruitment procedures described above have been successful in attracting an equal number of male and female participants from varied socioeconomic backgrounds and geographic locations, they have been insufficient in recruiting a diverse pool with respect to race and ethnicity. One of our goals is to hire a Community Outreach and Engagement Manager to help us increase the diversity of the participant pool through community partnerships.

Retaining participants who have enrolled in the Registry is also a sustained, time-intensive activity. We have found that continuity of research personnel is a critical factor for supporting continued enrollment. Participants vary in terms of the time commitment they can make to our research studies. The Research Coordinator plays a critical role in establishing rapport and connections with participants and monitoring the number of research requests. To maintain the connection with participants and keep them engaged in our research, we send out (*via* mail and email) quarterly newsletters, where we can inform participants about ongoing and upcoming studies, report recent findings, introduce new team members, or share lab accomplishments. Retaining participants also depends on the quality of their experience with the research team while completing the studies. We have protocols for interacting with research participants that include professional dress, maintaining clear professional boundaries, and what to do if a participant reports an acute medical or psychological event. The overwhelming majority of our research team has clinical training as well as significant training and experience in working with individuals with TBI and brings this knowledge with them when engaging with participants from the Registry. Teams with less clinical expertise or less experience conducting patient-based research would require extensive training in such protocols.

Some attrition of research participants is expected given unexpected life events, changes in health status, availability, or relocation. Our efforts to retain participants have been highly effective: our retention rate is 99%. Despite this success in retaining participants, compared to our experiences with focal lesion patients, we have found that retaining participants with TBI to be more difficult and labor intensive, with larger gaps in participation, compared to focal lesion participants. A number of characteristics common in TBI may account for these differences. First, focal lesion participants are, on average, older (e.g., stroke is a common etiology for lesion studies) and thus less likely to be moving for economic (jobs, school) reasons. Second, the very nature of the behavioral consequences of TBI, leading to a range of poor outcomes, can make it difficult for some individuals to participate regularly or long-term. In our interactions with our participants, we have seen first hand the struggles individuals with TBI can have responding to changing life circumstances (e.g., housing insecurity, loss of employment). Repeated assessment and interactions inherent in the Registry provide us a clearer window into the lived experiences of individuals with TBI than we have when conducting single-timepoint group studies.

We recruit non-injured comparison participants using institutional mass emails, social media, community fliers, and word of mouth. Recruitment is driven by our needs for close demographic matching at the level of the individual or group and is determined by the demographics of enrolled participants with TBI. Recruitment of non-injured comparison participants can be just as challenging as TBI recruitment, particularly for certain demographics. For example, many of our participants with TBI are male and have 12 or fewer years of education. Recruiting non-injured men with 12 or less years of education has proven challenging, but we have had increased success by working with a digital marketing group to help us tailor our materials and strategies to reach target demographics. This example points to a secondary benefit of the Registry: in recruiting participants who demographically match adults with TBI, we have developed a parallel, large sample of diverse (e.g., across age and education) non-injured comparison participants that can make unique contributions to the basic science literature in psychology and cognitive neuroscience, where many studies rely on recruitment of undergraduate students (Gallander Wintre et al., 2001).

### Accessibility

There are barriers to participating in research studies and when unacknowledged or unaddressed our studies fail to represent, and sample across, the full range of experiences and outcomes following TBI. For example, participant schedules vary considerably as a function of outcome. Participants who have returned to work full time require evening and weekend sessions, while others are unemployed or work part time and have more flexibility during the week. Fatigue is an important consideration for scheduling sessions with participants who work full time for an evening session and we consider that when making decisions about specific tasks. Another barrier to participation is access to transportation. We are increasingly including compensation for transportation expenses (e.g., Uber, gas, or bus fare money) into our grants to reduce this barrier. In addition, the nature of cognitive deficits in TBI can make it difficult to navigate a large medical center, including parking and finding the research lab. We have developed materials with visual (maps) and written instructions to send to participants in advance of their visit and meet them in easy-to-find locations to walk with them to the lab. Facilities that offer valet parking for research participants can reduce cognitive load and stress by removing the need to find and remember the location of a parking spot. We also tailor our communications regarding session confirmations, reminders, and rescheduling to the individual preferences and needs of the participants (e.g., email, text messages). This process is guided by our team’s speech-language pathologists, who have extensive experience in scaffolding participation in clinical and research activities (e.g., with checklists and reminders at set intervals).

With the start of the COVID 19 pandemic, we moved many of our studies online and conducted remote data collection sessions. The need to temporarily stop in-person research visits was an opportunity to rethink accessibility barriers and to extend the geographical reach of the research participants we study. Before the pandemic, we only recruited individuals who were physically able to come to the lab. When we moved many of our studies online, we were able to recruit individuals with significant mobility challenges and participants from across the country in both rural and urban settings. We also created a laptop loan program for participants interested in participating but who did not have the necessary hardware. For one study, we mailed all study materials (e.g., headphones, actigraphy monitors, schedules, and checklists) to participants with prepaid return shipping labels (Morrow et al., 2022). There are, of course, many studies that must be conducted in person (e.g., neuroimaging), but our ability to extend Registry enrollment beyond our local community or state has allowed our studies to be more accessible and increases the diversity of our participant pool.

### Data Management

Registry data is managed using REDCap (Harris et al., 2009), a HIPPA-compliant and secure web application, in which data are stored in a secure MySQL database. REDCap allows for data entry from paper forms (e.g., neuropsychological assessments) and also elicited directly from participants in the form of REDCap-implemented surveys. Use of REDCap allows for secure storage of Registry data (access to Registry data is restricted to members of the research team by username and password), but also flexible and organized access (selected measures can be quickly de-identified and downloaded in multiple file formats).

### Reproducibility

Another benefit of the registry model is the opportunity to establish patient registries at multiple institutions with a core set of shared data elements. Based on training and work with the Iowa and Vanderbilt Registries, the senior author is establishing the Trajectories after Brain Injury Patient Registry at the University of Minnesota. This Registry will collect a set of core data elements (demographic data, neuropsychological characterization) in common with the Vanderbilt Registry, facilitating the combination of data sets and allowing for even larger-scale studies in the future. Efforts to sample across all levels of variability in individuals with TBI are strengthened by geographic and institutional diversity: while both Registries seek to recruit diverse samples in their own local contexts, differences in clinical partnerships and other local factors are likely to skew samples toward a particular subset of individuals with TBI. Aggregating data from across these differing local contexts will help to increase the diversity of our samples. Commitment to large sample sizes necessarily requires collaborative work and the development of infrastructure to support data-sharing and data aggregation will be critical for meeting the challenges of decomposing heterogeneity in this population.

### Summary

Developing and maintaining a patient registry modeled after registries in cognitive neuroscience and extended to meet the specific challenges of studying TBI is feasible. The research infrastructure provided by a patient registry offers a methodological tool and resource for improving our understanding of interindividual differences in behavioral profiles and functional outcomes as well as the development of interventions to improve long-term outcomes. In the next section, we describe some unique future research directions that are facilitated by a registry model.

## Analytic Approaches and Future Opportunities for Discovery Afforded by a Registry Model

The availability of a large number of richly characterized research participants afforded by a patient registry allows for the design of studies that can capitalize on multidimensional data in different ways, depending on the study’s goal. Clustering approaches cluster individuals into subgroups based on their similarity to one another across multiple dimensions. These subgroups can be meaningful: In a study of adults with autism, an initial group-level deficit (Baron-Cohen et al., 2015) was found to be driven by a minority of participants with significant impairments, a subgroup uncovered using clustering analyses (Lombardo et al., 2016). Another approach made possible by large, multidimensional datasets is normative modeling, which allows for statistical inference at the level of the individual, moving away from traditional group-mean focused analyses (Marquand et al., 2016, 2019). In intervention studies, the deep description of neuropsychological profile, neuroanatomical characterization, and demographic information, that we describe above, offers the opportunity to explore treatment moderators (see Morrow et al., 2021; Morrow et al., in revision). These potential moderators can be selected *a priori*, based on prior research or theory, but the registry approach we describe also allows for the potential to develop subgroups of participants who are similar in ways we can’t easily predict because of the multilevel factors at play. As a concrete example, heterogeneous responses to behavioral intervention have been demonstrated in a treatment study of adults with binge-eating disorder (Sysko et al., 2010). Participants were clustered into subgroups prior to intervention, and were randomly assigned to one of three treatment strategies. Two subgroups demonstrated improvements regardless of treatment approach, while the remaining subgroups only improved under a single treatment approach.

Patient registries of individuals with TBI also confer a number of unique future opportunities to link and track behavioral and structural changes associated with aging and long-term outcomes. For example, both healthy and pathological aging are associated with specific and well-documented neuroanatomical changes, but little is known about the interaction between the neuropathological sequelae of TBI and the aging process or the risk for aberrant aging. Registries are well positioned to conduct longitudinal studies which confer methodological advantages for examining change over time (Lindenberger and Pötter, 1998; Hofer and Sliwinsky, 2001; Lindenberger et al., 2011). Furthermore, registries can help facilitate high-quality, longitudinal studies that can extend the power of emerging neuroimaging techniques, from identifying patients with TBI at cross-section to identify, and 1 day predict, subgroups of individuals at risk for poor long-term outcomes. This type of research agenda fits with recent calls for improved and expanded data collection that will guide more precise and tailored clinical management of TBI across the lifespan (Haarbauer-Krupa et al., 2021).

Finally, patient registries can provide a critical test bed for hypothesis driven research on precision rehabilitation, a long-term goal of our Registry. In the context of clinical-translational research, registries offer the critical infrastructure for studies that assess how participants respond to targeted interventions over extended periods of time (e.g., even years post-injury or while living in the community), which is critical in determining which interventions enact meaningful change in chronic disability. More specifically, registries could support research that tests the efficacy of different types of interventions (behavioral, pharmacological, neuromodulatory) using designs that take into account treatment moderators, drawn from multidimensional registry data (e.g., demographic, neuropsychological, neuroanatomical). Longitudinal data collection can be facilitated by technologies that are accessible and acceptable to individuals with TBI (e.g., smartphones, actigraphy) for remote, continuous monitoring and treatment of a range of post-injury symptoms (e.g., mood, fatigue, pain, cognition). TBI patient registries stand poised to capitalize on the inherent heterogeneity of TBI and can uniquely serve to determine which individual characteristics matter in predicting outcome, acting as a keystone in the arc of research to design targeted, precision interventions that reduce long-term disability for all patients with TBI (Covington and Duff, 2021; Morrow et al., 2022).

## Conclusion

Behavioral and functional outcomes following TBI are highly variable. Heterogeneity in outcome is a barrier to development and delivery of personalized treatment protocols for the specific and unique deficit profile of a given patient, and impedes our ability to predict outcomes across levels of analysis and across time. We propose that patient registries inspired by those in cognitive neuroscience, which support the recruitment of large representative samples and the collection of multidimensional and longitudinal data, can meet many challenges that are common in TBI research. A registry approach provides a powerful tool to better characterize multilevel sources of heterogeneity, and promises to lead to new discoveries regarding the identification of factors that influence individual differences in behavioral and functional outcomes in TBI, including those that may also be critical moderators in recovery and treatment efficacy. Finally, the registry approach may benefit participants themselves, providing them insight into their deficits, and to the research process more broadly. For example, one the participants in the Registry wrote us to share what he feels he gained through his participation:

*“I feel like the real benefits of your program are lost on the patient when presented as a ‘study.’ I have gained a lot out of these studies as they have challenged me, and have helped build confidence in the status of my brain. I wish there was better way to ‘market’ to patients that this is really to help them understand where they are. I know there is a myriad of legal reasons for sure why this study can’t be called ‘therapy’ or ‘rehab’ but I am convinced the more individuals who would participate the better off they would be.”*

A registry approach, with frequent and sustained interactions over time with individuals with TBI, who are the critical stakeholders of our research, allows participants to directly inform the direction of the research program and to be at the center of the effort to improve long-term outcomes following TBI.

## Author Contributions

MD and NC planned the scope and content of the review. MD, EM, ME, RM, and NC did the majority of the writing for the initial version of the manuscript with assistance from SC. All authors contributed to the final version of the manuscript, in content and in the writing and editing.

## Conflict of Interest

The authors declare that the research was conducted in the absence of any commercial or financial relationships that could be construed as a potential conflict of interest.

## Publisher’s Note

All claims expressed in this article are solely those of the authors and do not necessarily represent those of their affiliated organizations, or those of the publisher, the editors and the reviewers. Any product that may be evaluated in this article, or claim that may be made by its manufacturer, is not guaranteed or endorsed by the publisher.
